# Identification and Functional Analysis of Key Autophosphorylation Residues of Arabidopsis Senescence Associated Receptor-like Kinase

**DOI:** 10.3390/ijms23168873

**Published:** 2022-08-09

**Authors:** Zhaoxia Guo, Yuanyuan Mei, Dan Wang, Dong Xiao, Xianglin Tang, Yaru Gong, Xinxin Xu, Ning Ning Wang

**Affiliations:** Tianjin Key Laboratory of Protein Sciences, College of Life Sciences, College of Agricultural Sciences, Nankai University, Tianjin 300071, China

**Keywords:** senescence-associated receptor-like kinase AtSARK, autophosphorylation, reversible protein phosphorylation, senescence-suppressed protein phosphatase SSPP, leaf senescence, Arabidopsis

## Abstract

Reversible protein phosphorylation mediated by protein kinases and phosphatases plays important roles in the regulation of leaf senescence. We previously reported that the senescence-associated leucine-rich repeat receptor-like kinase AtSARK autophosphorylates on both serine/threonine and tyrosine residues and functions as a positive regulator of Arabidopsis leaf senescence; the senescence-suppressed protein phosphatase SSPP interacts with and dephosphorylates the cytoplasmic domain of AtSARK, thereby negatively regulating leaf senescence. Here, 27 autophosphorylation residues of AtSARK were revealed by mass spectrometry analysis, and six of them, including two Ser, two Thr, and two Tyr residues, were further found to be important for the biological functions of AtSARK. All site-directed mutations of these six residues that resulted in decreased autophosphorylation level of AtSARK could significantly inhibit AtSARK-induced leaf senescence. In addition, mutations mimicking the dephosphorylation form of Ser384 (S384A) or the phosphorylation form of Tyr413 (Y413E) substantially reduced the interaction between AtSARK and SSPP. All results suggest that autophosphorylation of AtSARK is essential for its functions in promoting leaf senescence. The possible roles of S384 and Y413 residues in fine-tuning the interaction between AtSARK and SSPP are discussed herein.

## 1. Introduction

Leaf senescence is a highly regulated developmental process, ultimately leading to leaf death. Accompanied by ordered disassembly of macromolecules that reallocates nutrients to other newly formed organs, leaf senescence has evolved as a life history strategy critical for plant fitness and environmental adaptation [1]. The tight regulation of leaf senescence is complex and involves many signals from both inside the plant and the outside environments [2]. Although substantial progress has been made in addressing the underlying molecular mechanism of leaf senescence, the manner by which the senescence signals are transduced to regulate the initiation and progression of leaf senescence remains elusive.

Reversible protein phosphorylation, orchestrated by protein kinases and phosphatases, is a central mechanism in the regulation of signal transduction [3]. Receptor-like kinases (RLKs) are a large family of protein kinases having a unique structure. A typical RLK generally consists of an extracellular receptor domain, a transmembrane helix, and a cytoplasmic kinase domain followed by a carboxyl-terminal tail (CT) [4]. The receptor domain is critical in perceiving the extracellular signals, whereas the kinase domain functions in transmitting the signals into the cell to activate the downstream regulatory pathway via phosphorylation. In the model plant *Arabidopsis thaliana*, genome-wide analysis revealed 610 RLKs classified into 44 subfamilies, among which leucine-rich repeat receptor-like protein kinases (LRR-RLKs) represent the largest subfamily, with more than 200 members [5]. Autophosphorylation is a critical event for the activation of RLK and downstream signaling responses [6,7]. Plant RLKs predominantly catalyze phosphorylation of serine and threonine residues on target proteins. The bona fide tyrosine kinases are absent in plant genomes. Nevertheless, Ser/Thr and Tyr dual-specificity protein kinases with the capability to phosphorylate Tyr residues were reported in plants [8]. It has been suggested that tyrosine phosphorylation exerts major regulatory effects on the dual-specificity RLK-mediated signaling initiation and transduction in plants [9].

Plant RLKs play crucial roles in the regulation of leaf senescence. For example, receptor-like protein kinase 1 (RPK1), an LRR-RLK in Arabidopsis, positively regulates both natural and ABA-mediated leaf senescence [10]. OsSIK2, a S-domain RLK in rice, negatively regulates dark-induced leaf senescence [11]. Another LRR-RLK member in Arabidopsis, somatic embryogenesis receptor kinase 4 (SERK4), was characterized as a negative regulator of leaf senescence [12]. More recently, a cell wall-associated receptor-like kinase in Arabidopsis, AtWAKL10, was also found to negatively regulate leaf senescence [13]. SARK, the abbreviation for senescence-associated receptor-like kinase, represents a class of receptor-like kinases related to senescence. Thus, one can imagine that different SARKs might exert their effects on leaf senescence by different modes of action. For example, the *PpSARK* gene in *Physcomitrella patens* acts as a negative regulator of senescence [14]. However, *PvSARK* regulates leaf senescence positively in *Phaseolus vulgaris* L [15]. *AtSARK* in Arabidopsis and its homolog *GmSARK* in soybean, which were previously characterized by our group, function as positive regulators of leaf senescence [16,17].

Protein phosphatases function as balancing switches to reverse kinase action through removal of phosphates from target proteins. Dephosphorylation by protein phosphatases often renders protein kinases inactive and thereby suppresses their cellular functions [18]. For example, the *Pseudomonas syringae*-secreted phosphatase HopAO1 can target the G-type lectin receptor-like kinase lipooligosaccharide-specific reduced elicitation (LORE) in Arabidopsis for tyrosine dephosphorylation, and thus suppress plant immune responses [19]. Emerging evidence has shown that protein phosphatases also play important roles in the regulation of leaf senescence. For example, *AtPAP26*, a PP2C-type protein phosphatase encoding gene, was up-regulated in senescing leaves of Arabidopsis [20]. The T-DNA insertion mutant *atpap26* showed delayed leaf senescence [21]. Overexpression of *SAG113*, another PP2C-type protein phosphatase encoding gene in Arabidopsis, led to precocious leaf senescence, while knockout of this gene resulted in delayed leaf senescence [22]. A type 2A protein phosphatase PP2A-B’γ was recently found to negatively regulate the onset of developmental leaf senescence in Arabidopsis and the *pp2a-b′γ* mutant exhibited premature leaf senescence in the apical parts of leaves [23]. Nevertheless, few key protein phosphatases that pair with known protein kinases to coordinately regulate leaf senescence have been characterized.

Both AtSARK and GmSARK mentioned above encode Ser/Thr and Tyr dual-specificity LRR-RLKs and positively regulate leaf senescence through the synergistic actions of auxin and ethylene [16,17]. We further found that senescence-suppressed protein phosphatase (SSPP), a PP2C-type protein phosphatase, negatively regulates leaf senescence by interacting with and dephosphorylating the cytoplasmic domain of AtSARK [24]. However, the autophosphorylation sites of AtSARK and the effects of autophosphorylation on its biological functions and its interaction with SSPP remain uncharacterized.

In this study, 27 autophosphorylation residues in the cytoplasmic domain of AtSARK were identified by mass spectrometry analysis. Six of them, including two Ser, two Thr, and two Tyr, were found to be important for the autophosphorylation activity of AtSARK by site-directed mutagenesis and in vitro autophosphorylation assay. In addition, all the site-directed mutations that reduced the autophosphorylation activity of AtSARK significantly inhibited AtSARK-induced premature leaf senescence, suggesting that the catalytic activity and phosphorylation status of AtSARK is crucial for its biological function in promoting leaf senescence. Furthermore, we demonstrated that two of the six residues, S384 and Y413, may play important but distinct roles in the interaction between AtSARK and SSPP.

## 2. Results

### 2.1. Identification of Key Autophosphorylation Residues of Senescence-Associated Receptor-like Kinase AtSARK

**Figure 1:**
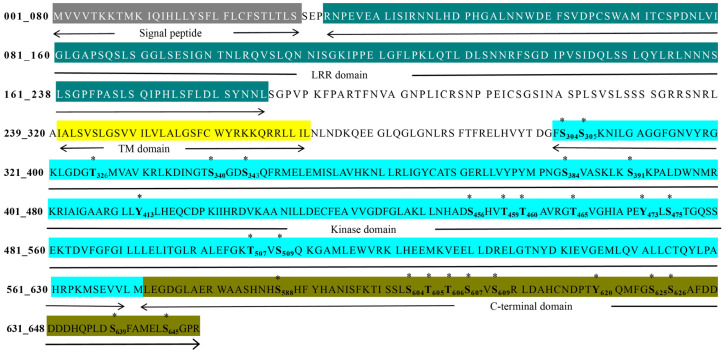
Predicted autophosphorylation sites of *Arabidopsis thaliana* senescence-associated receptor like kinase AtSARK by liquid chromatography-tandem mass spectrometry (LC-MS/MS). The signal peptide, leucine rich repeat (LRR) domain, transmembrane (TM) domain, kinase domain, and C-terminal domain of full-length AtSARK protein are highlighted in different colors and marked with the solid line below the sequence. All 27 potential autophosphorylation residues of AtSARK predicted by LC-MS/MS are shown in bold letters and indicated with stars (*).

The previous study of our group showed that LRR-RLK AtSARK functions as a positive regulator of leaf senescence. AtSARK possesses dual specificity and can auto-phosphorylate on both Ser/Thr and Tyr residues in vitro [16]. To further identify the potential autophosphorylation residues, the bacterially expressed cytoplasmic kinase domain of AtSARK fused with glutathione S-transferase (GST-AtSARK-CD) was purified by affinity chromatography, and in vitro autophosphorylation reaction was performed as described previously [16]. The fully phosphorylated GST-AtSARK-CD proteins were then subjected to liquid chromatography-tandem mass spectrometry (LC-MS/MS) analysis. The results revealed 27 autophosphorylation residues in AtSARK, which include 18 Ser, 6 Thr, and 3 Tyr residues (Figure 1). Among them, 16 residues were located in the kinase domain, namely, S304, S305, T326, S340, S343, S384, S391, Y413, S456, T459, T460, T465, Y473, S475, S507, and S509; the other 11 residues, namely, S588, S604, T605, T606, S607, S609, Y620, S625, S626, S639, and S645, are located in the C-terminal domain (Figure 1).

**Figure 2:**
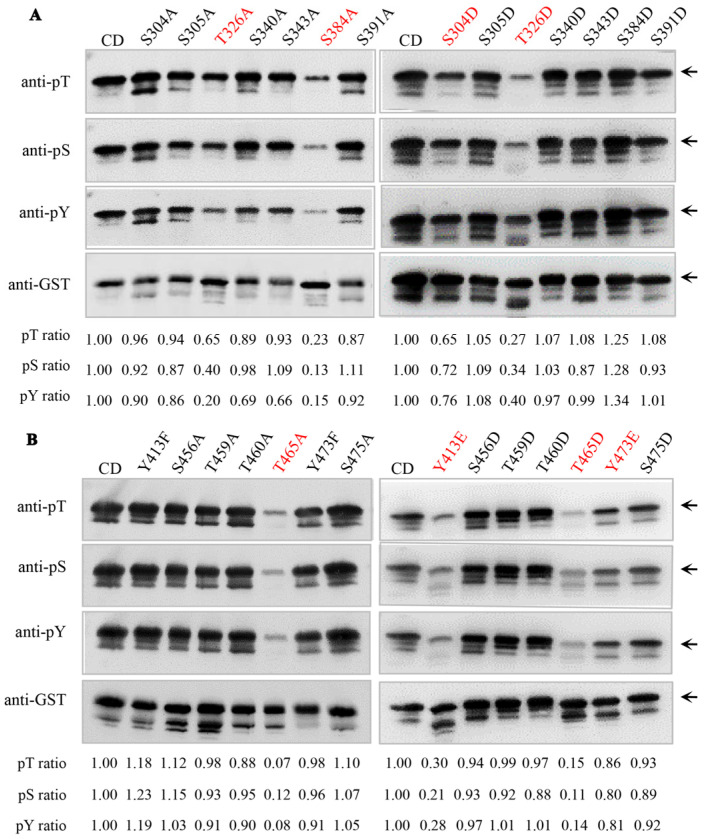
Identification of key autophosphorylation residues of AtSARK by site-directed mutagenesis and Western blot analysis. Each of the potentially phosphorylated Ser and Thr residues identified by LC-MS/MS was replaced with either an alanine (**A**), which cannot be phosphorylated, or an aspartic acid (D), which mimics phosphorylation. Each of the Tyr residues was substituted with either a phenylalanine (F) to mimic dephosphorylation or a glutamic acid (E) to mimic phosphorylation. The site-directed mutant proteins were subjected to in vitro autophosphorylation assay as described in Section 4. Autophosphorylation level was evaluated by Western blotting with anti-phospho-Ser (anti-pS), anti-phospho-Thr (anti-pT), and anti-phospho-Tyr (anti-pY) antibodies. The wild-type or mutated AtSARK-CD protein loaded in each lane was examined using anti-GST antibody. The molecular weight of AtSARK-CD protein is 72 kDa and phosphorylated bands of interests in each blot are indicated with black arrows. Numbers below the blots indicate the intensity ratio of the anti-pS, anti-pT, or anti-pY band to the anti-GST band of the indicated mutant protein with those of the wild-type AtSARK-CD protein normalized to 1. The results of site-directed mutants of S304, S305, T326, S384, and S391 residues are shown in panel (**A**), while those of Y413, S456, T459, T460, Y473, and S475 residues are shown in panel (**B**). The mutants with significantly reduced autophosphorylation level are highlighted in red. Data shown are the typical results obtained from three independent biological replicates. CD, GST-AtSARK-CD.

Phosphorylation adds a negative charge to amino acid side chains. Substituting a phosphorylatable site (e.g., Ser, Thr or Tyr) to the negatively charged residue Asp (D) / Glu (E) or the positively charged residue Ala (A) / Phe (F) represents a classic and frequently used approach to mimic the phosphorylation or dephosphorylation status of that residue, respectively. To further examine the effects of phosphorylation status on the kinase activity of AtSARK, we replaced each of the Ser and Thr residues identified above with either A or D and mutated each of the Tyr residues to either E or F to mimic the different phosphorylated forms of these residues. After purification by affinity chromatography, the site-directed mutant proteins of GST-AtSARK-CD were subjected to in vitro autophosphorylation assay and then compared with the wild-type AtSARK-CD protein for autophosphorylation activity by Western blotting with anti-phospho-Ser, anti-phospho-Thr, and anti-phospho-Tyr antibodies, respectively (Figure 2 and Figure S1). The results revealed six key residues for the autophosphorylation of AtSARK, namely, two Ser, two Thr, and two Tyr. As shown in Figure 2A, phosphorylation-mimicking mutation of S304 residue, S304D, markedly inhibited autophosphorylation of AtSARK on serine, threonine, and tyrosine residues. The dephosphorylation-mimicking S384A mutant also showed decreased autophosphorylation levels, but the S384D mutant-mimicking phosphorylation at this residue exhibited enhanced autophosphorylation. Significantly reduced autophosphorylation activities were observed when the two Thr residues T326 and T465, in particular T465, was each mutated to either an alanine or an aspartic acid (Figure 2A,B). The phosphorylation-mimicking mutations of two Tyr residues, Y413E and Y473E with glutamic acid in place of tyrosine, both resulted in a great decrease in the autophosphorylation status of AtSARK (Figure 2B). However, the autophosphorylation activity of the dephosphorylation-mimicking Y413F mutant was slightly elevated, whereas that of Y473F did not differ from the wild-type AtSARK (Figure 2B).

### 2.2. The Autophosphorylation of AtSARK Is Essential for Its Function in Promoting Leaf Senescence

**Figure 3:**
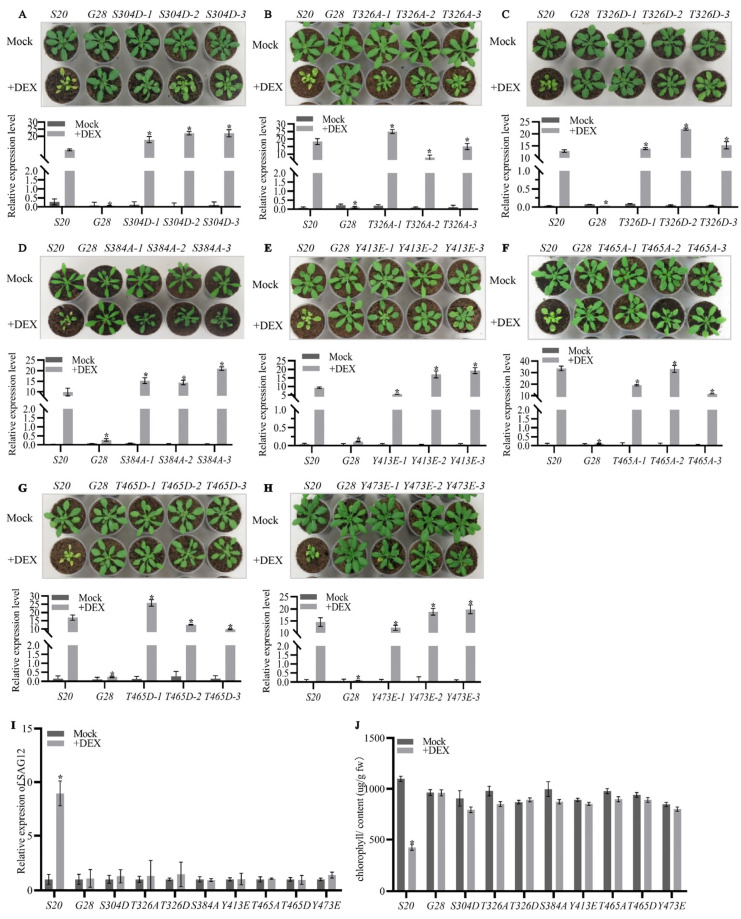
The autophosphorylation of AtSARK is essential for its function in promoting leaf senescence. Three independent lines of 19-d-old transgenic Arabidopsis overexpressing the indicated site-directed mutant of *AtSARK* under the control of *GVG* promoter were daily sprayed with dexamethasone (DEX) or its mock solvent as described in Section 4 and the photographs were taken after an additional 7 d (upper, **A**–**H**). The *GVG:AtSARK* (*S20*) and *GVG:GUS* (*G28*) plants were used as positive and negative control, respectively. Relative transcript levels of the exogenous wild-type or mutated *AtSARK* gene in each transgenic line were evaluated by real-time quantitative RT-PCR (qPCR) using *TIP41-Like* as internal control (lower, **A**–**H**). Data are means ± standard errors (SE) of three biological replicates. Stars indicate significant differences from DEX-treated *S20* plants (student *t*’-test, α = 0.05). (**I**) Transcript levels of the senescence-associated marker gene *SAG12* in the fifth and six leaves of each transgenic line after DEX or mock treatment were determined at 24 h. (**J**) Chlorophyll contents in the fifth and sixth leaves of indicated transgenic plants were measured following the method described in materials and methods. Data are means ± SE of three biological replicates. Stars indicate significant differences from mock-treated control plants (student *t*’-test, α = 0.05).

To further examine the effects of autophosphorylation on the biological functions of AtSARK, we generated transgenic Arabidopsis overexpressing all the above-mentioned site-directed AtSARK mutants that had altered autophosphorylation levels under the control of a dexamethasone (DEX)-inducible promoter (*GVG*, [25]). The homozygous *GVG:GUS* transgenic Arabidopsis (*G28*, [16]) was taken as the transformation control, while transgenic Arabidopsis overexpressing the wild-type *AtSARK* (*S20*, [16]) was used as a positive control. Transcript levels of the wild-type or mutated *AtSARK* genes in all the transgenic Arabidopsis were examined by quantitative RT-PCR. For each site-directed *AtSARK* mutant, three independent lines that exhibited similar transcript levels of the mutated *AtSARK* to that of the wild-type *AtSARK* in *S20* were selected for further analysis (Figure 3).

As described previously [16], the *S20* plants exhibited significantly early leaf senescence after DEX treatment. Nevertheless, all site-directed mutants of AtSARK with decreased autophosphorylation level, including the phosphorylation-mimicking mutants *S304D, T326D, T465D, Y413E*, and *Y473E*, and dephosphorylation-mimicking mutants *T326A, S384A*, and *T465A*, resembled *G28* and the mock-treated control plants, and did not show precocious leaf senescence phenotypes after DEX treatment (Figure 3A–H). Consistently, we found that the transcript level of senescence marker gene *SAG12* was significantly induced upon DEX treatment in *S20* plants. However, in the site-directed AtSARK mutants that had reduced levels of autophosphorylation, the transcript levels of *SAG12* did not change significantly after DEX treatment compared to those in the mock-treated control (Figure 3I). In addition, the chlorophyll content in the fifth and sixth rosette leaves of DEX-treated *S20* was significantly reduced but no such significant difference was observed in the site-directed mutants that had decreased autophosphorylation levels of AtSARK. All results suggest that the kinase activity of AtSARK is essential for its function in promoting leaf senescence.

### 2.3. S384 and Y413 Play Important Roles in the Interaction between AtSARK and Senescence-Suppressed Protein Phosphatase SSPP

**Figure 4:**
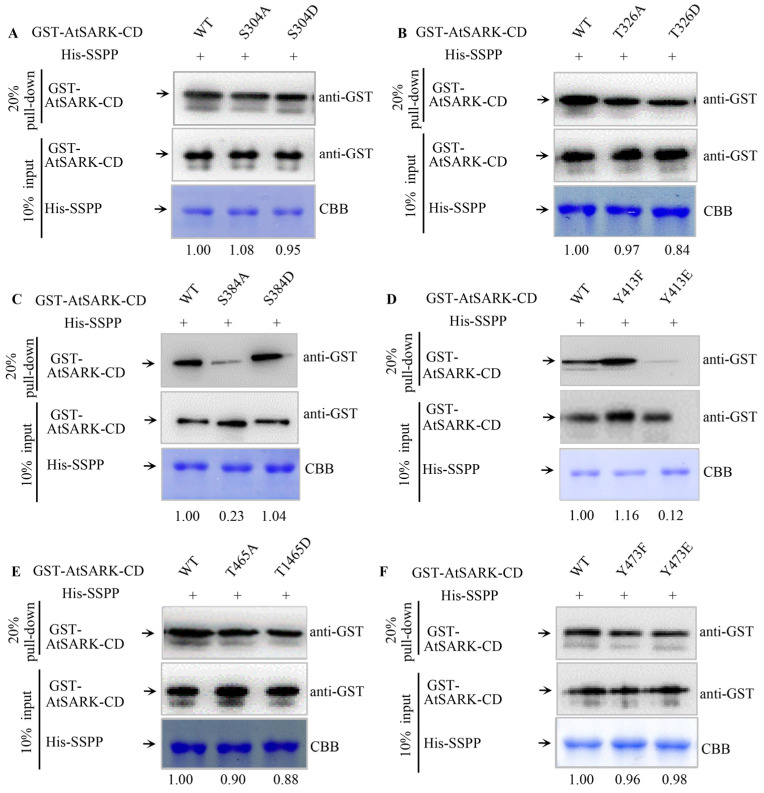
Impacts of phosphorylation status of six key phosphorylation residues of AtSARK on the interaction with senescence-suppressed protein phosphatase SSPP. (**A**–**F**): In vitro pull-down assays showing the interactions between SSPP and the phosphorylation- or dephosphorylation-mimicking mutants of AtSARK at S304, T326, S384, Y413, T465, and Y473 residues, respectively. GST-tagged wild-type AtSARK-CD (WT) or its mutated alternative were incubated with His-SSPP-bound Ni-NTA beads. Detailed protocol of the in vitro pull-down assay is described in Section 4. Input wild-type or mutated AtSARK proteins were detected with anti-GST antibody while input His-SSPP proteins were examined with Coomassie Brilliant Blue (CBB) staining. Solid arrows indicate the migration of each protein. Numbers below the blots indicate the relative intensity ratio between AtSARK pull-down band to input His-SSPP band in each lane with those of the wild-type AtSARK-CD normalized to 1. Data presented are the typical results of three independent biological replicates.

As we previously reported, the senescence-promoting function of AtSARK can be suppressed by SSPP through dephosphorylation of its cytoplasmic domain [24]. To further analyze the effects of AtSARK autophosphorylation status on its interaction with SSPP, in vitro pull-down assay was performed using site-directed phosphorylation- or dephosphorylation-mimicking mutants of AtSARK-CD at S304, T326, S384, Y413, T465, and Y473 residues, respectively. Interaction between SSPP and the wild-type AtSARK-CD protein was used as control. It was found that changes in phosphorylation status of S304, T326, T465, or Y473 residues did not affect the interaction between AtSARK and SSPP (Figure 4A,B,E,F). However, dephosphorylation-mimicking mutation of S384 residue, S384A, significantly inhibited the interaction of AtSARK with SSPP, although the interaction between phosphorylation-mimicking mutants S384D and SSPP did not differ from the wild-type control (Figure 4C). In contrast, Y413E, the phosphorylation-mimicking mutation of Y413, resulted in drastically abolished interaction. Y413F mutation that mimics dephosphorylation of this residue appeared to enhance the interaction between AtSARK and SSPP (Figure 4D). These observations suggest that S384 and Y413 are important for the interaction between AtSARK and SSPP.

**Figure 5:**
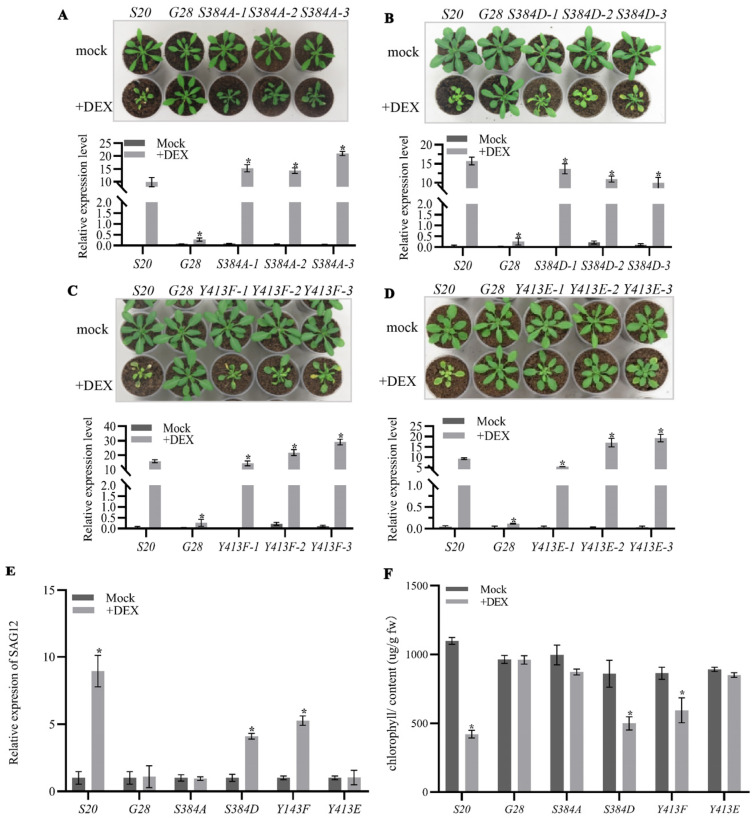
S384 and Y143 residues play distinct roles in AtSARK-mediated leaf senescence. Three independent lines of 19-d-old transgenic Arabidopsis overexpressing site-directed phosphorylation- or dephosphorylation-mimicking mutant of *S384* or *Y413* under the control of *GVG* promoter were daily sprayed with DEX or mock solution as described in Section 4. *GVG:AtSARK* (*S20*) and *GVG:GUS* (*G28*) transgenic plants were used as positive and negative control, respectively. Photographs were taken 7 days after treatment (**A**–**D**, upper). Relative transcript levels of exogenous wild-type or mutated *AtSARK* (**A**–**D**, lower) were evaluated by qPCR using *TIP41-like* gene as internal control. Data are means ± SE of three biological replicates. Stars indicate significant differences from DEX-treated *S20* plants (Student’s *t*’-test, α = 0.05). (**E**) Transcript levels of the senescence-associated marker gene *SAG12* in the fifth and six leaves of each transgenic line after DEX or mock treatment were determined at 24 h. (**F**) Chlorophyll contents in the fifth and sixth leaves of indicated transgenic plants were measured as detailed in Section 4. Data are means ± SE of three biological replicates. Stars indicate significant differences from mock-treated control plants (Student’s *t*’-test, α = 0.05).

We further examined the effects of changes in phosphorylation status of S384 and Y413 residues on the biological function of AtSARK. Transgenic Arabidopsis overexpressing site-directed phosphorylation- or dephosphorylation-mimicking mutant of *S384* or *Y413* under the control of DEX-inducible *GVG* promoter were generated. The above-mentioned *G28* and *S20* were used as a negative and positive control, respectively. Transcript levels of the exogenous *AtSARK* genes in all transgenic Arabidopsis, either the wild-type or the mutated form, were measured by quantitative RT-PCR. For each site-directed mutant, three independent lines, in which the transcript levels of mutated *AtSARK* were similar to the wild-type *AtSARK* in *S20*, were selected and used for further analysis (Figure 5). It was found that transgenic Arabidopsis overexpressing mutated *AtSARK* that had reduced interaction with SSPP, such as *S384A* and *Y413E*, did not show precocious leaf senescence after DEX treatment, resembling *G28* and the mock-treated control (Figure 5A,D). In contrast, transgenic Arabidopsis overexpressing *S384D* or *Y413F*, which showed a similar or higher level of interaction with SSPP, exhibited significantly accelerated leaf senescence compared to the positive control plants *S20* (Figure 5B,C). In line with this, transcripts of *SAG12* were significantly up-regulated in the DEX-treated *S384D* and *Y413F* mutants, and in the *S20* control. Nevertheless, no such increase was detected in the DEX-treated *S384A* and *Y413E* mutants (Figure 5E). Similarly, chlorophyll contents were significantly reduced in *S384D* and *Y413F* mutants after DEX treatment; however, those in *S384A* and *Y413E* mutants did not differ significantly from their mock-treated control counterparts (Figure 5F).

## 3. Discussion

Protein kinase and phosphatase-mediated reversible protein phosphorylation plays a pivotal role in cellular signaling. In plants, protein phosphorylation is predominantly achieved by RLKs [5]. Many plant RLKs can achieve their optimal kinase activity only after autophosphorylation of specific residues, which facilitates recruitment of downstream interacting proteins [26]. Identification of autophosphorylation sites of a kinase protein represents a crucial step in understanding its biochemical characteristics and delineating the signaling network with which it is involved.

We previously identified the dual-specificity receptor-like protein kinase AtSARK as a positive regulator of leaf senescence [16], and further, the PP2C type protein phosphatase SSPP was found to negatively regulate leaf senescence by interacting with and dephosphorylating AtSARK [24]. To gain more insights into the molecular mechanism of SARK-induced leaf senescence and the interaction between AtSARK and SSPP, autophosphorylation sites of AtSARK were identified and functionally characterized in this study.

### 3.1. Autophosphorylation of AtSARK Is Required for Its Functions in Promoting Leaf Senescence

In total, 27 autophosphorylation sites in the cytoplasmic domain of AtSARK were identified by LC-MS/MS analysis (Figure 1). Among them, six residues in the kinase domain were found to be important for the autophosphorylation of AtSARK, which consist of two Ser, two Thr, and two Tyr (Figure 2). Mutating these phosphorylation sites had strikingly different effects on the autophosphorylation activity and biological functions of AtSARK (Figure 2 and Figure 3). However, all site-directed mutations that reduced the autophosphorylation level of AtSARK significantly inhibited AtSARK-induced premature leaf senescence (Figure 3). These results suggest that the kinase activity and high autophosphorylation level of AtSARK are important for its biological function in promoting leaf senescence.

It is noteworthy that the effects of phosphorylation-mimicking mutations of these six residues on the autophosphorylation status and biological function of AtSARK vary greatly. For example, the S384D mutation that mimics phosphorylation of S384 resulted in elevated autophosphorylation activity of AtSARK, whereas substitution of S384 residue with A to mimic dephosphorylation substantially suppressed the autophosphorylation level of AtSARK (Figure 2). Transgenic Arabidopsis overexpressing the *S384D* mutant showed precocious leaf senescence, whereas those overexpressing the *S384A* mutant did not differ from *G28* control (Figure 5). By contrast, phosphorylation-mimicking mutations of S304, Y413, and Y473 residues all reduced the autophosphorylation levels of AtSARK and suppressed AtSARK-induced leaf senescence (Figure 2 and Figure 3). These observations suggest the existence of a delicate mechanism that fine-tunes the initiation and progression of leaf senescence. Given the special structure of AtSARK as a typical receptor-like kinase, we assume that AtSARK can perceive and transmit extracellular signals into the cell to activate and autophosphorylate its cytoplasmic kinase domain. The phosphate group(s) in the AtSARK-CD domain then will be transferred to the downstream senescence-associated targets to further amplify the senescence signal. Thus, the precise autophosphorylation control of AtSARK through key residues exerts profound effects on its biological functions in the regulation of leaf senescence.

Phosphorylation of the conserved threonine in the activation loop is often required for full activity of many protein kinases [27]. In this study, markedly abolished autophosphorylation activity was observed when the conserved T465 residue in the activation loop of AtSARK was mutated to either an alanine or an aspartic acid (Figure 2). Consistently, compared to *S20* control plants that overexpressed wild-type *AtSARK*, leaf senescence was significantly suppressed in the transgenic Arabidopsis overexpressing *T465A* or *T465D* mutants (Figure 3). These results support the notion that the conserved Thr residue in the activation loop of an RLK is essential for its kinase activity. Interestingly, significantly lower autophosphorylation activity was also observed, albeit to a less extent, when T326 was replaced with either an alanine or an aspartic acid. This observation highlights the importance of T326 for the autophosphorylation activity and biological function of AtSARK. Given the fact that T326 is located in the kinase domain but does not fall into the activation loop region, the detailed function of T326 residue deserves further analysis.

Tyrosine phosphorylation of dual-specificity RLK has emerged as a fundamentally important mechanism controlling plant growth, development, and innate immunity [28]. As shown in Figure 2 and Figure 3, phosphorylation-mimicking mutations of both Tyr residues of AtSARK, Y413E and Y473E, substantially reduced the autophosphorylation level of AtSARK and inhibited AtSARK-induced leaf senescence. The results imply that phosphorylation of these two Tyr residues plays a negative role in the autophosphorylation and biological function of AtSARK, but the detailed mechanism remains unclear. The spatiotemporal dynamics of phosphorylation of these two Tyr residues and the possible mechanism underlying the senescence signal-induced inhibition of Tyr phosphorylation of AtSARK are also largely unknown. An in-depth study will help to understand how leaf senescence is initiated and progressed in a timely and appropriate manner.

### 3.2. The Phosphorylated S384 Residue of AtSARK Is the Main Dephosphorylation Site for SSPP Whereas the Phosphorylation of Y413 Residue Fine-Tunes the Interaction between AtSARK and SSPP

We previously reported that AtSARK and SSPP functions as a protein kinase–phosphatase pair with counteracting effects on the regulation of leaf senescence. During this process, AtSARK cannot phosphorylate SSPP but acts as its substrate [24]. This implies that SSPP can only interact with and catalyze the dephosphorylation of phosphorylated AtSARK and thereby suppresses its biological function. In this study, the effects of phosphorylation status of AtSARK on its interaction with SSPP were analyzed by in vitro pull-down assay using site-directed phosphorylation- or dephosphorylation-mimicking mutagenesis at the aforementioned six key residues of AtSARK, namely, S304, T326, S384, Y413, T465, and Y473. Among all the site-directed mutants that had decreased autophosphorylation activity, two mutants, namely, S384A and Y413E, showed significantly reduced interaction with SSPP (Figure 4C,D), implying that S384 and Y413 residues play important roles in the interaction between AtSARK and SSPP.

Given the fact that the dephosphorylation-mimicking mutation of AtSARK at S384, S384A, led to impaired interaction with SSPP (Figure 4C), we propose that the phosphorylated S384 residue may be the main site of dephosphorylation by SSPP. Dephosphorylation by SSPP at S384 would render AtSARK inactive and thus lose its ability to promote leaf senescence. Consistent with this hypothesis, we found that transgenic Arabidopsis overexpressing the *S384A* mutant of *AtSARK* under the *GVG* promoter did not show precocious leaf senescence after DEX treatment (Figure 5). Notably, not all site-directed mutations that mimicked dephosphorylation and resulted in a decreased autophosphorylation level of AtSARK impaired the interaction between AtSARK and SSPP. With the exception of S384A, the dephosphorylation-mimicking mutations of the other two residues, namely, T326A and T465A, also led to a significantly decreased autophosphorylation level of AtSARK but did not affect the interaction between AtSARK and SSPP, further confirming that the phosphorylated S384 residue represents one of the main dephosphorylation sites for SSPP.

Unlike S384, for the Y413 residue, it was the phosphorylation-mimicking mutation, Y413E, that weakened the interaction between AtSARK and SSPP (Figure 4D). Similarly, the phosphorylation-mimicking mutant Y413E, rather than the dephosphorylation-mimicking mutant Y413F, exhibited markedly reduced autophosphorylation activity of AtSARK and lost the ability to promote leaf senescence (Figure 2 and Figure 5). We speculate that the phosphorylated Y413 residue may be the dephosphorylation site for another unknown protein phosphatase instead of SSPP. The reversible phosphorylation of Y413 residue may be involved in fine-tuning the progression of leaf senescence via modulating the interaction between AtSARK and SSPP at S384. However, the biological significance of this hypothesis remains to be established in more detailed studies.

Taken together, the current study identified six key phosphorylation residues of AtSARK. Phosphorylation mimicking mutations of these residues exerts different effects on the kinase activity, autophosphorylation level, and biological functions of AtSARK, implying that modulation on the autophosphorylation status of AtSARK plays a pivotal role in the regulation of leaf senescence. In particular, identification of S384 and Y413 residues as important sites for the interaction between AtSARK and SSPP suggests that the initiation and progression of leaf senescence orchestrated by AtSARK and SSPP involves more complex and tight regulation.

It is noteworthy that *AtSARK*-overexpressing Arabidopsis also displayed growth suppression in addition to precocious leaf senescence. As considerable cross-talks exist between senescence and stresses responses, we cannot rule out the possibility that AtSARK might be also involved in stress responses. However, we tend to assume that *AtSARK* plays more important roles in controlling natural leaf senescence, based on the observations that AtSARK is a natural senescence up-regulated RLK [16] and the overexpression of *AtSARK* results in significantly elevated expression of *SAG12* (Figure 3 and Figure 5)*,* a frequently used marker gene specifically activated by age-dependent senescence [29]. Further investigations of the upstream components, such as the ligand of AtSARK, will contribute to better understanding of the initial steps for the activation of AtSARK-mediated senescence signaling.

## 4. Materials and Methods

### 4.1. Liquid Chromatography-tandem Mass Spectrometry (LC–MS/MS) Analysis and Phosphosite Identification

The in vitro protein autophosphorylation experiments were slightly modified from a previous report [16]. A total of 100 μg of the purified GST-AtSARK-CD protein was incubated with 10 μM ATP in the phosphorylation buffer (25 mM Tris-HCl, pH 7.5, 10 mM MgCl_2_, 10 mM MnCl_2_, 1 mM dithiothreitol) at 30 °C for 20 min. LC–MS/MS analysis of phosphorylation sites was carried out by BGI-tech, China. Phosphopeptide identification was performed using an in-house Mascot Server (version 2.3.01, Matrix Science Inc., Boston, USA), and data were interrogated using the SwissProt database, NCBI nonredundant protein database, and the EST divisions of EMBL database. The parameter settings allowed trypsin digestion for a maximum of one missed cleavage site and fixed modifications of carbamidomethyl. Gln->pyro-Glu (N-term Q), oxidation (M), phospho (ST) and phospho (Y) were considered as variable modifications. Peptide mass tolerances were set up to 15 parts per million (ppm), and fragment mass tolerance was set to 20 million mass units (mmu). Phosphorylation sites were validated by manual inspection of MS/MS spectra with predicted fragments.

### 4.2. Site-Directed Mutagenesis of AtSARK and Recombinant Protein Purification

The site-specific mutations of the cytoplasmic region of AtSARK (AtSARK-CD) were generated using the Fast Mutagenesis System Kit (Transgene, Beijing, China) following the manufacturer’s instructions. Primers used for each site-directed mutagenesis are listed in Table S1. The amplified fragments of *AtSARK-CD* mutants were subcloned into pMD18-T vector and inserted into pGEX-6p-1 vector after verification by sequencing. The GST-AtSARK-CD proteins with and without mutation were expressed in *Escherichia coli* Rosetta 2 (DE3) plysS and purified by affinity chromatography using a Glutathione Sepharose 4B column (GE Healthcare, Buckinghamshire, UK) according to the manufacturer’s recommended protocol. The recombinant His-SSPP protein was generated as described previously [24] and purified by affinity chromatography using Ni^2+^NTA agarose columns (GE Healthcare, Buckinghamshire, UK).

### 4.3. In Vitro Autophosphorylation Assay

The in vitro protein autophosphorylation experiments were performed essentially as reported previously [16], with minor modifications. A total of 1 μg of the purified GST-AtSARK-CD protein or its mutated alternative was incubated with 10 μM ATP in the phosphorylation buffer (25 mM Tris-HCl, pH 7.5, 10 mM MgCl_2_, 10 mM MnCl_2_, 1 mM dithiothreitol) at 30 °C for 20 min. The phosphorylation reactions were stopped by adding 5×SDS-PAGE loading buffer and boiling for 10 min. Immunoblot analysis was carried out to detect the in vitro phosphorylation activities of the purified protein with anti-PT/anti-PY antibody (Cell Signaling Technology, Beverly, MA, USA), anti-PS antibody (GE Healthcare Life Sciences, Chicago, IL, USA), and anti-GST (Cell Signaling Technology, Beverly, MA, USA), respectively, following the manufacturer’s instructions of the Tanon-5500 chemiluminescent imaging system (Tanon, Shanghai, China).

### 4.4. Pull-Down Assay

The in vitro pull-down assay was performed as described previously [30]. Briefly, Ni Sepharose 6 Fast Flow beads (GE Healthcare, Buckinghamshire, UK) bound with His-SSPP were washed with Binding buffer (1.5 M NaCl, 10.1 mM Na_2_HPO_4_, 1.8 mM KH_2_PO_4_, and 20 mM imidazole). To each reaction, approximately 10 μg GST-AtSARK protein with or without mutation as determined by Bradford reagent method on a Multiskan GO plate reader (Thermo scientific, Vantaa, Finland) was added. Subsequently, reaction mixtures were incubated for at least 2 h at 4 °C with gentle rotation. Proteins were eventually eluted with 100 μL elution buffer (1.5 M NaCl, 10.1 mM Na_2_HPO_4_, 1.8 mM KH_2_PO_4_, and 500 mM imidazole) after washing at least five times and boiled with 5 × SDS-PAGE loading buffer for 10 min. The proteins were separated by 12% SDS-PAGE for Coomassie Brilliant Blue (CBB) staining and immunodetection with anti-GST antiserum (Cell Signaling Technology, Beverly, MA, USA) at 1:2000 dilutions. The images were scanned and recorded by a Tanon-5500 chemiluminescence imaging system (Tanon, Shanghai, China). Data shown are representative of at least three independent experiments.

### 4.5. Plant Growth and Generation of Transgenic Lines

*Arabidopsis thaliana* ecotype Col-0 was used as the wild-type and conditions for plant growth were as described previously [24]. The site-specific mutations of AtSARK were generated using the Fast Mutagenesis System Kit (Transgene, Beijing, China). All primers used are listed in supplemental Table S1. After being confirmed by DNA sequencing, each mutated fragment of interest was inserted into the pTA7002 vector and introduced into *Agrobacterium tumefaciens* strain GV3101. Transformation was performed on wild-type Arabidopsis plants using the floral dip method [31]. Transformants were screened on 1/2 × MS medium containing 15 mg L^−1^ hygromycin, and the resistant seedlings were transferred to soil. Homozygous single-copy T3 plants were used for all experiments. In order to analyze the phenotype of adult plants, the 19-day-old soil-grown *GVG:AtSARK* transgenic plants or its mutated alternative were daily sprayed with 30 μM dexamethasone (DEX) or a simulated solution containing 0.01% Tween 20 (mock) for 3 d. The *GVG:GUS* plants (*G28*) were used as transformation control. Photographs were taken at 10 d by a Canon PowerShot G16 (Canon Inc., Tokyo, Japan). For each transgenic line, seven plants were tested in one biological replicate. Three independent biological replicates were performed, and typical results are shown.

### 4.6. RNA Extraction and RT-PCR Analysis of Gene Expression

The fifth and sixth leaves of 19-d-old transgenic plants sprayed with DEX or its mock solution were harvested at 24 h to check the expression levels of *AtSARK* and the senescence-associated marker gene *SAG12*. RNA extraction and cDNA synthesis were undertaken as described previously [16]. Quantitative RT-PCR analysis was performed using the SYBR Green Perfect mix (TaKaRa, Dalian, China) on an iQ5 (Bio-Rad, California, USA) machine following the manufacturer’s instructions. All reactions were carried out under the following conditions: 95 °C for 5 min; and 40 cycles of 95 °C for 30 s and 57 °C for 30 s. The *TIP41-like* gene was used as an internal control. Three independent biological replicates were performed. All primers used in RT-PCR analysis were designed to the exon junctions and are listed in Supplemental Table S1.

### 4.7. Determination of Chlorophyll Content

The chlorophyll content in the fifth and sixth leaves of transgenic Arabidopsis was spectrophotometrically measured at 7 d after three-day consecutive DEX or mock treatment as described previously [32]. At least three independent samples were examined, all of which produced the typical results shown in this article.

## Data Availability

Not applicable.

## Supplementary Materials

The following supporting information can be downloaded at: https://www.mdpi.com/article/10.3390/ijms23168873/s1.
