# Supplementary material for: Identification and Functional Analysis of Key Autophosphorylation Residues of Arabidopsis Senescence Associated Receptor-like Kinase
Source: Int J Mol Sci. 2022 Aug 9;23(16):8873. doi: 10.3390/ijms23168873 (PMC9408895; doi:10.3390/ijms23168873)
Supplement: Supplementary file 1 [file ijms-23-08873-s001.zip › Table S1.pdf]

Table S1. Sequences of the primers used in this study.

Name	Primer sequence (5′-3′)
S304A F	CATGTTTATACAGATGGTTTCGCTTCCAAGAACA
S304A R	GCGAAACCATCTGTATAAACATGGAGTTCTCT
S304D F	TGTTTATACAGATGGTTTCGATTCCAAGAA
S304D R	TCGAAACCATCTGTATAAACATGGAGTTCTCT
S305A F	TTATACAGATGGTTTCAGTGCCAAGAACAT
S305A R	CACTGAAACCATCTGTATAAACATGGAGTTCT
S305D F	TTATACAGATGGTTTCAGTGACAAGAACATT
S305D R	TCACTGAAACCATCTGTATAAACATGGAGTTCT
T326A F	GAGGCAAGCTTGGAGATGGGGCAATGGTGGC
T326A R	CCCCATCTCCAAGCTTGCCTCTGTACACATT
T326D F	GAGGCAAGCTTGGAGATGGGGACATGGTGGCAG
T326D R	GTCCCCATCTCCAAGCTTGCCTCTGTACACATT
S340A F	TGAAGGATATTAATGGAACCGCAGGGGATTCA
S340A R	CGGTTCCATTAATATCCTTCAACCGTTTCACT
S340D F	TGAAGGATATTAATGGAACCGACGGGGATTCACA
S340D R	GTCGGTTCCATTAATATCCTTCAACCGTTTCACT
S343A F	TTAATGGAACCTCAGGGGATGCACAGTTTCGT
S343A R	CATCCCCTGAGGTTCCATTAATATCCTTCAACCGT
S343D F	TTAATGGAACCTCAGGGGATGACCAGTTTCGT
S343D R	GTCATCCCCTGAGGTTCCATTAATATCCTTCAACC
S384A F	CCCTTACATGCCTAATGGAGCCGTCGCCTCT
S384A R	GCTCCATTAGGCATGTAAGGGTAAACAAGAAG
S384D F	CCCTTACATGCCTAATGGAGACGTCGCCTCTA
S384D R	TCTCCATTAGGCATGTAAGGGTAAACAAGAAG
S391A F	GCGTCGCCTCTAAGCTTAAAGCTAAACCGGC
S391A R	CTTTAAGCTTAGAGGCGACGCTTCCATTAG
S391D F	GCGTCGCCTCTAAGCTTAAAGATAAACCGGC
S391D R	TCTTTAAGCTTAGAGGCGACGCTTCCATTAG
Y413E F	GTGCAGCGAGAGGTTTGTTGGAGCTACATGAGCA
Y413E R	CTCCAACAAACCTCTCGCTGCACCAATTGCTAT
Y413F F	GTGCAGCGAGAGGTTTGTTGTTTCTACATGAG
Y413F R	AACAACAAACCTCTCGCTGCACCAATTGCTAT
S456A F	AGCTCCTTAACCATGCGGATGCTCATGTCAC
S456A R	CATCCGCATGGTTAAGGAGCTTTGCGAGTCC
S456D F	AGCTCCTTAACCATGCGGATGATCATGTCACA
S456D R	TCATCCGCATGGTTAAGGAGCTGCCTTTACAT
T459A F	ACCATGCGGATTCTCATGTCGCAACTGCGGTC
T459A R	CGACATGAGAATCCGCATGGTTAAGGAGCTTTG
T459D F	CCATGCGGATTCTCATGTCGATACTGCGGTCCG
T459D R	ATCGACATGAGAATCCGCATGGTTAAGGAGCTT
T460A F	ATGCGGATTCTCATGTCACAGCTGCGGTCCGT

T460A R	CTGTGACATGAGAATCCGCATGGTTAAGGAGC
T460D F	TGCGGATTCTCATGTCACAGATGCGGTCCGTG
T460D R	TCTGTGACATGAGAATCCGCATGGTTAAGGAG
T465A F	TCACAACTGCGGTCCGTGGTGCGGTTGGCCAC
T465AR	CACCACGGACCGCAGTTGTGACATGAGAATCC
T465D F	CACAACTGCGGTCCGTGGTGACGTTGGCCACAT
T465D R	GTCACCACGGACCGCAGTTGTGACATGAGAATC
Y473F F	GTTGGCCACATTGCACCTGAATTTCTCTCCACTG
Y473F R	AATTCAGGTGCAATGTGGCCAACCGTACCACGGA
Y473E F	GTTGGCCACATTGCACCTGAAGAACTCTCCACT
Y473E R	TTCTTCAGGTGCAATGTGGCCAACCGTACCACGGA
S475A F	CACATTGCACCTGAATATCTCGCCACTGGTCA
S475A R	CGAGATATTCAGGTGCAATGTGGCCAACCGT
S475D F	CACATTGCACCTGAATATCTCGACACTGGTCAGT
S475D R	TCGAGATATTCAGGTGCAATGTGGCCAACCGT
T507A F	GAGAGCTCTTGAGTTTGGTAAAGCCGTTAGCCA
T507A R	CTTTACCAAACTCAAGAGCTCTCAGTCCGGT
T507D F	GAGAGCTCTTGAGTTTGGTAAAGACGTTAGCCAGA
T507D R	TCTTTACCAAACTCAAGAGCTCTCAGTCCGGT
S509A F	TCTTGAGTTTGGTAAAACCGTTGCCCAGAAAGGA
S509A R	GCAACGGTTTTACCAAACTCAAGAGCTCTCAGT
S509D F	TGAGTTTGGTAAAACCGTTGACCAGAAAGGA
S509D R	TCAACGGTTTTACCAAACTCAAGAGCTCTCAG
S588A F	TGGGCTGCTTCGCATAACCATGCACATTTCTA
S588A R	CATGGTTATGCGAAGCAGCCCATCTCTCGGCT
S588D F	TGGGCTGCTTCGCATAACCATGACCATTTCTACC
S588D R	CATGGTTATGCGAAGCAGCCCATCTCTCGGCT
S604A F	TCAAGACAATCTCTTCTCTGGCTACTACTTC
S604A R	CCAGAGAAGAGATTGTCTTGAAAGAGATATTG
S604D F	TCAAGACAATCTCTTCTCTGGATACTACTTC
S604D R	TCCAGAGAAGAGATTGTCTTGAAAGAGATATTG
T605A F	AGACAATCTCTTCTCTGTCTGCTACTTCTGTC
T605A R	CAGACAGAGAAGAGATTGTCTTGAAAGAGAT
T605D F	AGACAATCTCTTCTCTGTCTGATACTTCTGTC
T605D R	TCAGACAGAGAAGAGATTGTCTTGAAAGAGAT
T606A F	CAATCTCTTCTCTGTCTACTGCTTCTGTCTC
T606A R	CAGTAGACAGAGAAGAGATTGTCTTGAAAGAG
T606D F	CAATCTCTTCTCTGTCTACTGATTCTGTCTCA
T606D R	TCAGTAGACAGAGAAGAGATTGTCTTGAAAGAG
S607A F	TCTCTTCTCTGTCTACTACTGCTGTCTCAAG
S607A R	CAGTAGTAGACAGAGAAGAGATTGTCTTGAAAG
S607D F	TCTCTTCTCTGTCTACTACTGATGTCTCAAGGC
S607D R	TCAGTAGTAGACAGAGAAGAGATTGTCTTGAAAG
S609A F	CTCTGTCTACTACTTCTGTCGCAAGGCTTGAC

S609A R	CGACAGAAGTAGTAGACAGAGAAGAGATTGTC
S609D F	CTCTGTCTACTACTTCTGTCGATAGGCTTGACG
S609D R	ATCGACAGAAGTAGTAGACAGAGAAGAGATTGTC
Y620F F	CACATTGCAATGATCCAACTTTTCAAATGTTTG
Y620F R	AAAGTTGGATCATTGCAATGTGCGTCAAGC
Y620E F	ACATTGCAATGATCCAACTGAACAAATGTTTG
Y620E R	TTCAGTTGGATCATTGCAATGTGCGTCAAGCCTTG
S625A F	CAACTTATCAAATGTTTGGAGCTTCGGCTTTC
S625A R	CTCCAAACATTTGATAAGTTGGATCATTGCA
S625D F	CAACTTATCAAATGTTTGGAGATTCGGCTTTC
S625D R	TCTCCAAACATTTGATAAGTTGGATCATTGCA
S626A F	CTTATCAAATGTTTGGATCTGCGGCTTTCGA
S626A R	CAGATCCAAACATTTGATAAGTTGGATCATT
S626D F	CTTATCAAATGTTTGGATCTGACGCTTTCGATGA
S626D R	GTCAGATCCAAACATTTGATAAGTTGGATCATT
S639A F	ATGATCATCAGCCTTTAGATGCCTTTGCCATG
S639A R	CATCTAAAGGCTGATGATCATCGTCATCATC
S639D F	GATGATCATCAGCCTTTAGATGACTTTGCCATG
S639D R	TCATCTAAAGGCTGATGATCATCGTCATCATCG
S645A F	GATTCCTTTGCCATGGAACTAGCCGGTCCAAG
S645A R	CTAGTTCCATGGCAAAGGAATCTAAAGGCTG
S645D F	GATTCCTTTGCCATGGAACTAGACGGTCCAAGA
S645D R	TCTAGTTCCATGGCAAAGGAATCTAAAGGCTGATG
rtAtSARK F	TCCATCGACCAGCTAAGCAGCCT
rtAtSARK R	AGCAGGGAAGGGCCCAGACA
rtSAG12 F	GGATGTCCCGGTTAATGATG
rtSAG12 R	TGGAAATCAAAACCACCTCC
rtTIP F	GTATGAAGATGAACTGGCTGACAAT
rtTIP R	ATCAACTCTCAGCCAAAATCGCAAG
