# Supplementary figures and images for: Identification and Functional Analysis of Key Autophosphorylation Residues of Arabidopsis Senescence Associated Receptor-like Kinase
Source: Int J Mol Sci. 2022 Aug 9;23(16):8873. doi: 10.3390/ijms23168873 (PMC9408895; doi:10.3390/ijms23168873)

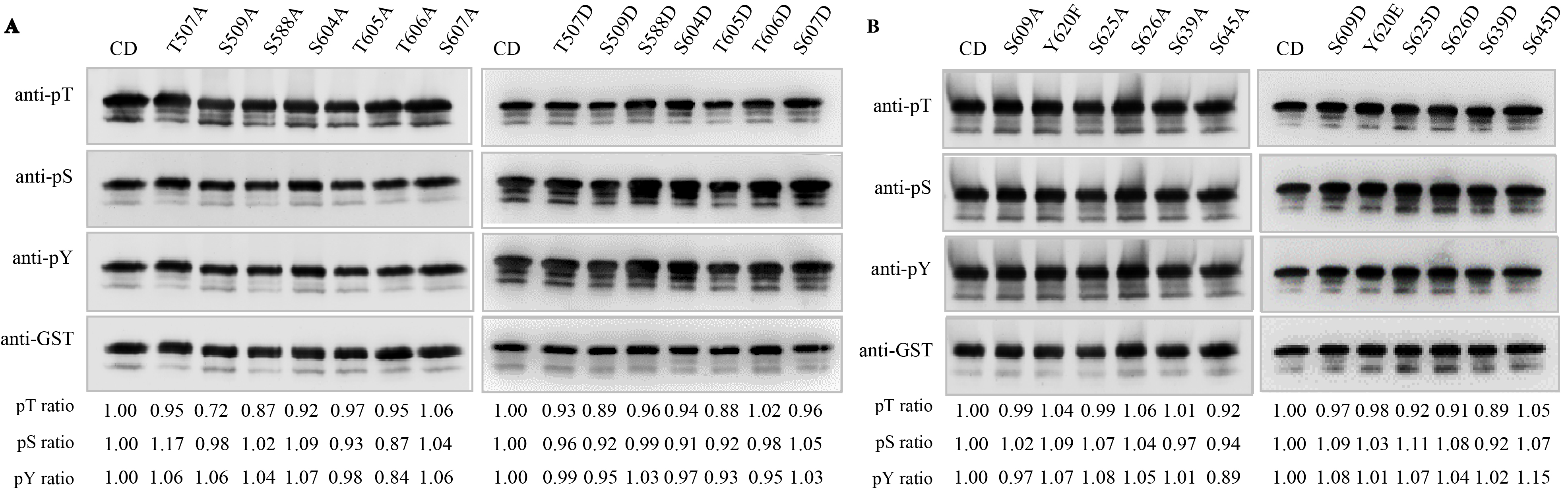

Supplement: Supplementary file 1 [file ijms-23-08873-s001.zip › Figure S1.jpg]
